# Crystal structure and spectroscopic analysis of a new oxalate-bridged Mn^II^ compound: *catena*-poly[guanidinium [[aqua­chlorido­manganese(II)]-μ_2_-oxalato-κ^4^ *O* ^1^,*O* ^2^:*O* ^1′^,*O* ^2′^] monohydrate]

**DOI:** 10.1107/S2056989016006605

**Published:** 2016-04-26

**Authors:** Hiba Sehimi, Ichraf Chérif, Mohamed Faouzi Zid

**Affiliations:** a Laboratoire de Matériaux et Cristallochimie, Faculté des Sciences de Tunis, Université de Tunis El Manar, 2092 Manar II Tunis, Tunisia; b Université de Gabès, Faculté des Sciences de Gabès, Campus Universitaire, Cité Erriadh Zrig, Gabès, 6072, Tunisia

**Keywords:** crystal structure, manganese, oxalate bridge, coordination polymer

## Abstract

As part of our studies on the synthesis and the characterization of oxalate-bridged compounds *M*–ox–*M* (ox = oxalate dianion and *M* = transition metal ion), we report the crystal structure of a new oxalate-bridged Mn^II^ phase, {(CH_6_N_3_)[Mn(C_2_O_4_)Cl(H_2_O)]·H_2_O}_*n*_. In the compound, a succession of Mn^II^ ions (situated on inversion centers) adopting a distorted octa­hedral coordination and bridged by oxalate ligands forms parallel zigzag chains running along the *c* axis. These chains are inter­connected through O—H⋯O hydrogen-bonding inter­actions to form anionic layers parallel to (010). Individual layers are held together *via* strong hydrogen bonds involving the guanidinium cations (N—H⋯O and N—H⋯Cl) and the disordered non-coordinating water mol­ecule (O—H⋯O and O—H⋯Cl), as well as by guanidinium π–π stacking. The structural data were confirmed by IR and UV–Visible spectroscopic analysis.

## Abstract 2

A novel oxalate-bridged manganese +II compound, *catena*-poly[guanidinium [[aqua­chlorido­manganese(II)]-μ_2_-oxalato-κ^4^ *O* ^1^,*O* ^2^:*O* ^1′^,*O* ^2′^] monohydrate], has been synthesized as single crystals at room temperature and characterized by X-ray diffraction, infrared and UV–Visible spectroscopic analyses, confirming the formation of a layered-type three-dimensional structure.

## Chemical context   

Much attention had been devoted to the coordination chemistry of oxalate (ox) anions due to the inter­esting structural features and physical properties they possess (Chérif *et al.*, 2011 ▸; Dridi *et al.*, 2013 ▸; Decurtins *et al.*, 1997 ▸). Oxalate anions have been demonstrated to be one of the most versatile bridging ligands for the construction of coordination polymers when combined with transition metal cations. Manganese(II) is a promising cation with possibilities of forming one-dimensional oxalato-based coordination polymers, as evidenced by reports describing the structures of several topologically similar Mn^II^–ox–Mn^II^ chains [see, for example, García-Couceiro *et al.* (2005 ▸) or Beznischenko *et al.* (2009 ▸)]. In those compounds, the oxalate-bridged manganese framework may be considered as a single-chain magnet based on the oxalate linker (*e.g.* Clemente-León *et al.*, 2011 ▸). In this work, we report the synthesis and crystal structure determination of a new oxalate-bridged coordination compound, {(CH_6_N_3_)[Mn(C_2_O_4_)Cl(H_2_O)]·H_2_O}_*n*_ (I).
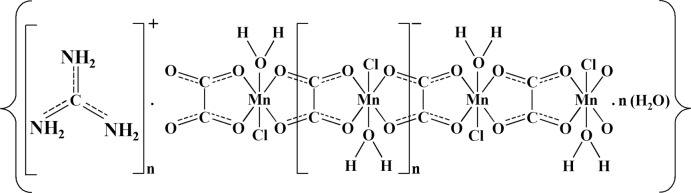


## Structural commentary   

**Figure 1:**
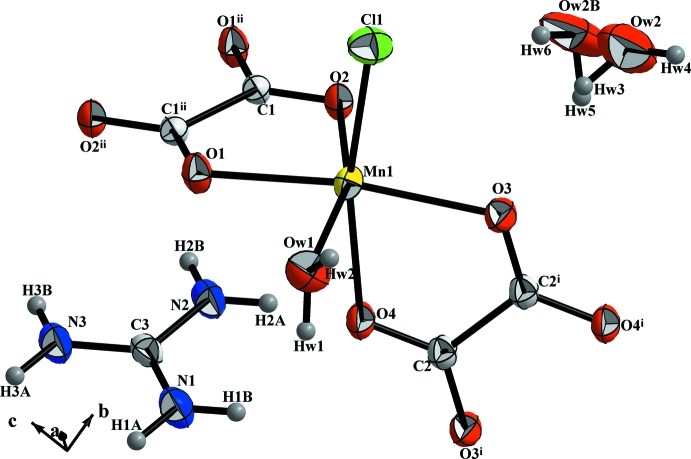
The structural unit of (I), showing the atom-numbering scheme. Displacement ellipsoids are drawn at the 50% probability level for non-H atoms. [Symmetry codes: (i) −*x* + 1, −*y* + 1, −*z* + 1; (ii) −*x* + 1, −*y* + 1, −*z*.]

The principal structural motifs of the title compound are the complex anion [MnCl(C_2_O_4_)(H_2_O)]^−^, the organic cation (CH_6_N_3_)^+^ and one disordered non-coordinating water mol­ecule. A bond-valence-sum calculation, assuming Mn—O and Mn—Cl bonds, gives a BVS value (Brown & Altermatt, 1985 ▸) of 2.05 (7), confirming the +II oxidation state of Mn and ensuring electrical neutrality of the formed unit. The coordination environment of the Mn^II^ ion involves two oxalate ligands exhibiting bis-chelating coordination modes, one chloride atom and one oxygen atom of the aqua ligand (Fig. 1 ▸) in a slightly distorted octa­hedral geometry. The small bite angles of the bis-chelating oxalate groups [73.99 (6)° for O3—Mn1—O4 and 75.35 (7)° for O1—Mn1—O2] and the extended Mn1—Cl1 bond [2.458 (2) Å] account for this distortion. The polyhedral distance and angle distortions, calculated from the Mn—O and Mn—Cl distances and O—Mn—O and O—Mn—Cl angles in the MnO_5_Cl unit, were found to be ID_d_ = 0.03 (2) and ID_a_ = 0.22 (4)%, respectively (Baur, 1974 ▸; Wildner, 1992 ▸).

The equatorial plane of the MnO_5_Cl octa­hedron is formed by atoms Mn1, O*W*1, O1, O2 and O3, with a calculated root-mean-square deviation of the fitted atoms of 0.1038 Å. The axial positions are occupied by the chloride atom [Mn1—Cl1 = 2.458 (2) Å] and one oxygen atom from the bridging oxalato group [Mn1—O4 = 2.248 (2) Å]. The two oxalato groups are almost perpendicular with a dihedral angle of 89.09 (6)°. The oxalate ion is located on an inversion center that also relates the two Mn atoms bonded to the oxalate ion with each other. The bridged metal ions are nearly coplanar with the oxalate plane with a mean deviation of 0.0147 (8) Å.

The Mn^II^ ion, as a *d* ^5^ high-spin system with a spherical electron distribution, has a limited number of commonly observed coordination geometries that are based on minimization of ligand–ligand repulsion. Among the Mn—O distances, the shortest are those involving an oxygen atom from the oxalate ion *trans* to another oxygen atom from the second oxalate ion. The range of these distances is 2.180 (1) to 2.194 (1) Å, which is in accord with those observed in other oxalate-bridged compounds such as one of the polymorphs of *catena*-poly[[di­aqua­manganese(II)]-μ-oxalato-κ^4^ *O* ^1^,*O* ^2^:*O* ^1′^,*O* ^2′^] (Soleimannejad *et al.*, 2007 ▸). The Mn—O distances involving the oxygen atoms of the oxalate ion *trans* to the coordinating water mol­ecule and *trans* to the chloride atom are slightly longer at 2.202 (2) and 2.248 (2) Å.

**Figure 2:**
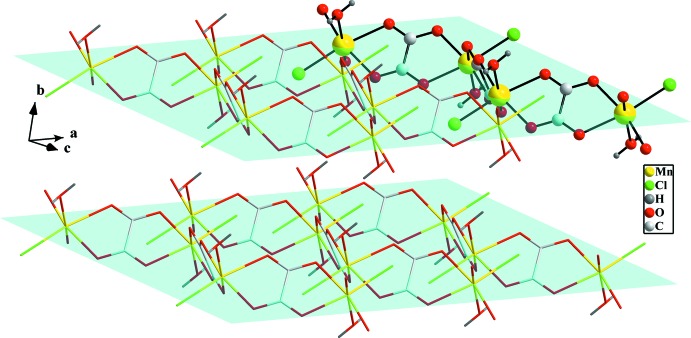
View of the structure packing showing Mn–Ox–Mn chains (highlighted by a ball-and-stick model) and layers parallel to (010) (blue planes).

The view of the structure packing (Fig. 2 ▸) shows the layered structure based on anionic zigzag oxalate-bridged Mn^II^ chains running along the *c* axis. The intra-chain Mn⋯Mn distances through bridging oxalate are 5.695 (2) and 5.778 (2) Å, somewhat longer than the value of 5.652 Å previously observed for {[Mn(C_2_O_4_)(C_8_H_7_N_3_)]·1.5H_2_O}_*n*_ (An & Zhu, 2009 ▸) involving a pyridyl-pyrazolide ligand instead of chloride and aqua ligands in the coordination environment of the Mn^II^ ion.

The geometric parameters for the guanidinium cations do not show any unusual features and are in agreement with those previously reported (Sakai *et al.* 2003 ▸; Vaidhyanathan *et al.*, 2001 ▸). The bond lengths [1.318 (2)–1.329 (2) Å] and angles [119.27 (16)–120.57 (16)°] are in the typical ranges, confirming a highly resonance-stabilized electronic structure and a completely delocalized charge between the three *sp* ^2^ nitro­gen atoms. Conjugation of the nitro­gen lone pairs with the empty *p*-orbital of the *sp* ^2^ carbon atom creates a planar cation.

## Supra­molecular features   

**Figure 3:**
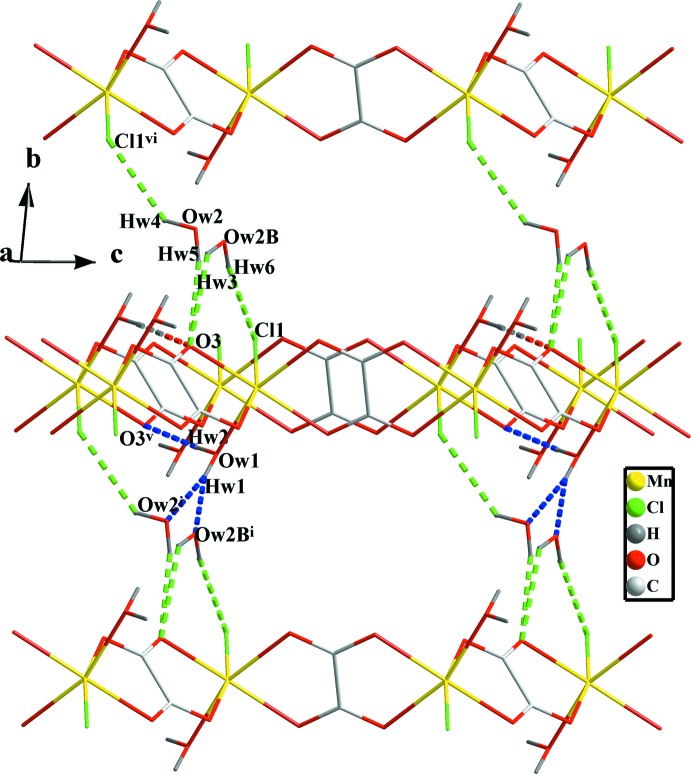
View of the hydrogen bonds developed by both coordinating (blue dashed lines) and non-coordinating (green dashed lines) water mol­ecules. [Symmetry codes: (i) *x*, *y* − 1, *z*; (v) −*x* + 2, −*y* + 1, -*z;* (vi) −*x* + 2, −*y* + 2, −*z*.]

Neighbouring oxalate-bridged zigzag chains are connected with each other *via* O—H⋯O hydrogen bonds involving the coordinating water mol­ecule. Its oxygen atom acts as a hydrogen-bond donor and establishes strong hydrogen bonds (Table 1 ▸) towards one of the oxalate oxygen atoms of a neighbouring chain (Fig. 3 ▸), O*W*1—H*W*2⋯O3^v^ [symmetry code: (v) −*x* + 2, −*y* + 1, −*z*], leading to the formation of anionic layers parallel to (010). A disordered non-coordin­ating water mol­ecule acts as acceptor (Fig. 3 ▸) for the other hydrogen atom involving the coordinating water mol­ecule *via* the hydrogen bonds O*W*1—H*W*1⋯O*W*2^i^ and O*W*1—H*W*1⋯O*W*2*B* ^i^ [symmetry code: (i) *x*, *y* − 1, *z*]. Both disorder components of the non-coordinating water mol­ecules act as hydrogen-bond donors towards oxygen atom O3 (Fig. 3 ▸) *via* the hydrogen bonds O*W*2—H*W*3⋯O3 and O*W*2*B*—H*W*5⋯O3, but they form different hydrogen bonds *via* their second H atom, to chlorine atoms in different lattice positions *via* hydrogen bonds O*W*2—H*W*4⋯Cl1^vi^ and O*W*2*B-*–H*W*6⋯Cl1 [symmetry code: (vi) −*x* + 2, −*y* + 2, −*z*]. The combined water hydrogen bonds link the anionic layers into a 3D framework.

**Figure 4:**
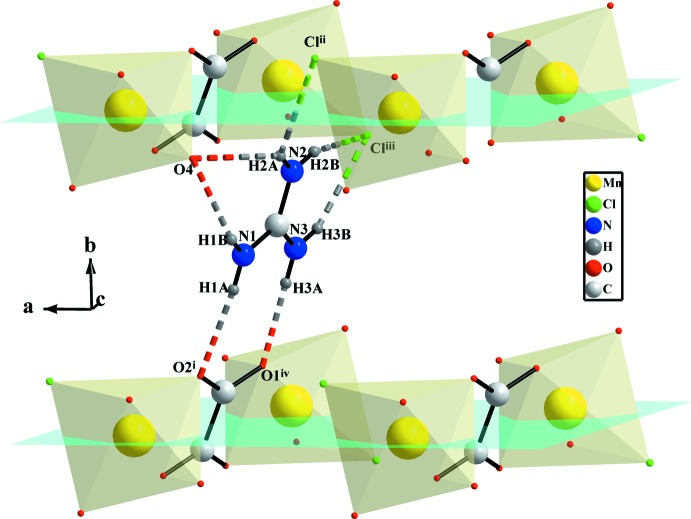
N—H⋯O and N—H⋯Cl hydrogen-bonding inter­actions developed by the guanidinium cations (dashed lines) in (I). Non-coordinating water mol­ecules and hydrogen atoms of coordinating water mol­ecules are omitted for clarity. [Symmetry codes: (i) *x*, *y* − 1, *z*; (ii) *x* − 1, *y*, *z*; (iii) −*x* + 1, −*y* + 1, −*z* + 1; (iv) −*x* + 1, −*y*, −*z* + 1.]

**Figure 5:**
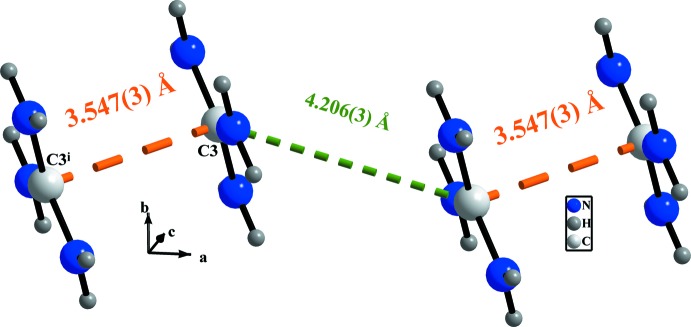
π–π stacking inter­actions (orange dashed lines) between adjacent organic cations. [Symmetry code: (i) −*x*, −*y*, −*z* + 1.]

The three N atoms of the guanidinium cation act as donors of hydrogen bonds N1—H1*A*⋯O2^i^, N1—H1*B*⋯O4, N2—H2*A*⋯Cl1^ii^, N2—H2*B*⋯Cl1^iii^, N2—H2*A*⋯O4, N3—H3*A*⋯O1^iv^ and N3—H3*B*⋯Cl1^iii^ [Table 1 ▸; symmetry codes: (i) *x*, *y* − 1, *z*; (ii) *x* − 1, *y*, *z*; (iii) −*x* + 1, −*y* + 1, −*z* + 1; (iv) −*x* + 1, −*y*, −*z* + 1], consolidating the anionic layers and giving additional stability to the three-dimensional structure as illustrated in Fig. 4 ▸. The guanidinium cations are also paired *via* π–π stacking with an inter­planar distance of 3.547 (3) Å between C3 and C3(−*x*, −*y*, −*z* + 1) (Di Tondo & Pritchard, 2012 ▸), as shown in Fig. 5 ▸.

## IR and UV–Vis characterizations   

**Figure 6:**
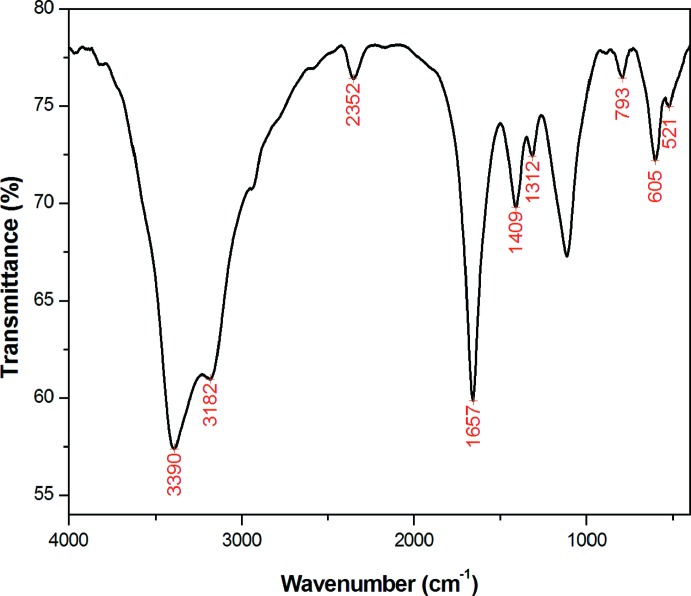
The IR spectrum of (I) in KBr.

The IR spectrum was recorded in the 4000–400 cm^−1^ region using a Perkin–Elmer spectrometer with the sample diluted in a pressed KBr pellet. The most intense IR absorption bands of (I) are given in Table 2 ▸. The spectrum (Fig. 6 ▸) displays broad and strong bands centered at 3390 and 3182 cm^−1^ assigned to [ν(O—H) + ν_as_(NH_2_)] and ν_s_(NH_2_), respectively (Sasikala *et al.*, 2015 ▸). The broadness of these bands is indicative of the presence of both coordinating and non-coordinating water mol­ecules, as well as –NH_2_ groups involved in an extensive hydrogen-bond framework, in agreement with the crystal structure. A weak band observed at 2352 cm^−1^ is attributed to an N—H⋯O stretching mode. The characteristic vibrations of the bridging oxalato ligand are observed at 1657 cm^−1^ [ν_as_(COO)], 1312 and 1409 cm^−1^ [ν_s_(COO)] and 793 cm^−1^ [δ(COO)] (Ma *et al.*, 2007 ▸). All these bands are consistent with the literature for a bis-chelating coordination of the oxalato ligand. Additional bands observed at around 605 and 521 cm^−1^ can be attributed to ν(Mn—Cl) (Zgolli *et al.*, 2011 ▸) and ν(Mn—O) (Biradar & Mruthyunjayaswamy, 2013 ▸), respectively.

**Figure 7:**
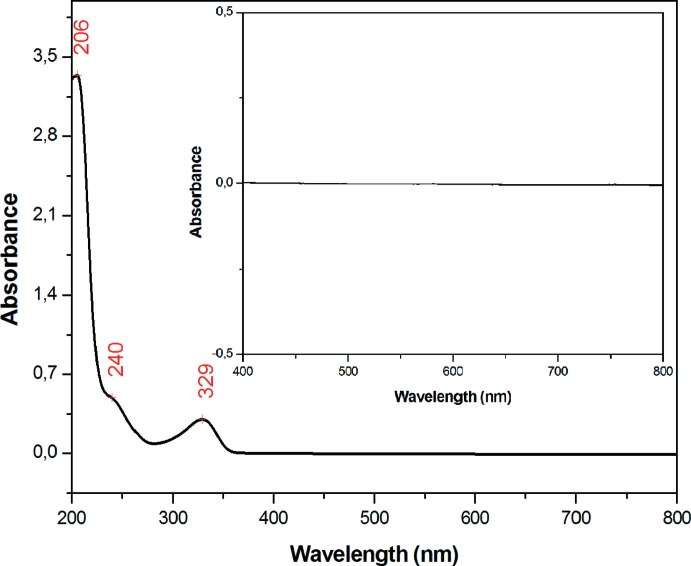
The UV–Vis spectrum of (I) in water. The insert is an expansion of the visible region.

Some crystals, selected under the microscope, were dissolved in 10 cm^3^ of distilled water. The solution obtained was analyzed using a UV–Visible spectrometer. The spectrum of (I) (Table 3 ▸ and Fig. 7 ▸) shows significant transitions at 206 nm (with a shoulder at 240 nm) and 329 nm. The first band is due to the π→π* transition of the guanidinium π system (Hoffmann *et al.*, 2009 ▸), the second witnesses the metal-to-ligand charge-transfer (Sun *et al.*, 1996 ▸) and the last corresponds to the *n*→π* transition (Sasikala *et al.*, 2015 ▸). An examination of the visible region of the spectrum does not reveal obvious *d*–*d* transitions (insert of Fig. 7 ▸) which may be too weak to be seen, as they are spin and Laporte forbidden, in accordance with the compound being almost colourless.

## Synthesis and crystallization   

Aqueous solutions of ammonium oxalate and guanidine hydro­chloride were added to Mn(SO_4_)·H_2_O dissolved in 10 cm^3^ of water in a 1:2:1 molar ratio. The resulting solution was left at room temperature and colourless crystals suitable for X-ray diffraction were obtained after two weeks of slow evaporation.

## Refinement   

Crystal data, data collection and structure refinement details are summarized in Table 4 ▸.

Guanidinium hydrogen atoms were positioned geometrically as riding atoms (N—H = 0.86 Å) using adequate HFIX instructions and refined with AFIX instructions. Hydrogen atoms of the coordinating water mol­ecule were found in Fourier difference maps. O—H distances were restrained to a value of 0.85 (1) Å and H⋯H distances were restrained to a value of 1.387 (1) Å.

The oxygen atom of the non-coordinating water mol­ecule had unusually high displacement parameters, and was refined as disordered over two alternative mutually exclusive positions. The solvent mol­ecule may be considered as being located vertically between negative-charged anionic layers formed by hydrogen-bonded polymeric chains and located horizontally between positive-charged pairs of guanidinium cations. This pseudo-channel affects its hydrogen-bonding inter­actions, see the discussion in the first paragraph of the *Supra­molecular features* section and Fig. 3 ▸, which may explain the observed disorder.

The disordered oxygen atom was refined as disordered over two positions O*W*2 and O*W*2*B* which were restrained to have similar geometries. Their hydrogen atoms were located from the Fourier difference maps. The O—H bond lengths were restrained to a value of 0.85 (1) Å and the H⋯H distances were restrained to a value of 1.387 (1) Å. The inter­atomic distances between the two pairs O*W*2 and H*W*5 and O*W*2*B* and H*W*3 were restrained to be equal using a SADI instruction with an effective standard deviation of 0.02. The hydrogen-bonding distance of hydrogen atom H*W*6 to chlorine atom Cl1 was restrained to 2.80 (1) Å. Subject to these and the above conditions, the occupancy ratio of the disordered non-coordinating water mol­ecule refined to 0.816 (13):0.184 (13).

## Supplementary Material

Crystal structure: contains datablock(s) I. DOI: 10.1107/S2056989016006605/zl2659sup1.cif

Structure factors: contains datablock(s) I. DOI: 10.1107/S2056989016006605/zl2659Isup2.hkl

CCDC reference: 1474882

Additional supporting information:  crystallographic information; 3D view; checkCIF report

## Figures and Tables

**Table 1:** Hydrogen-bond geometry (Å, °)

*D*—H⋯*A*	*D*—H	H⋯*A*	*D*⋯*A*	*D*—H⋯*A*
N1—H1*A*⋯O2^i^	0.86	2.19	3.047 (3)	175
N1—H1*B*⋯O4	0.86	2.20	2.917 (3)	140
N2—H2*A*⋯Cl1^ii^	0.86	2.85	3.509 (3)	135
N2—H2*B*⋯Cl1^iii^	0.86	2.56	3.357 (2)	154
N2—H2*A*⋯O4	0.86	2.38	3.054 (3)	135
N3—H3*A*⋯O1^iv^	0.86	2.00	2.854 (3)	172
N3—H3*B*⋯Cl1^iii^	0.86	2.57	3.363 (3)	154
O*W*1—H*W*1⋯O*W*2^i^	0.85 (1)	1.96 (1)	2.793 (4)	170 (3)
O*W*1—H*W*1⋯O*W*2*B* ^i^	0.85 (1)	1.82 (2)	2.643 (11)	165 (3)
O*W*1—H*W*2⋯O3^v^	0.84 (1)	2.06 (1)	2.890 (3)	176 (3)
O*W*2—H*W*3⋯O3	0.85 (1)	2.18 (2)	3.005 (5)	165 (5)
O*W*2—H*W*4⋯Cl1^vi^	0.85 (1)	2.61 (3)	3.319 (6)	142 (4)
O*W*2*B*—H*W*5⋯O3	0.85 (1)	2.42 (11)	2.840 (10)	112 (9)
O*W*2*B*—H*W*6⋯Cl1	0.86 (1)	2.81 (1)	3.656 (18)	172 (11)

Symmetry codes: (i) 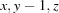; (ii) 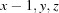; (iii) 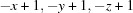; (iv) 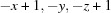; (v) 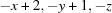; (vi) 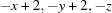.

**Table 2:** IR data (cm^−1^) for (I)

Wavenumber	Assignment
521	ν(Mn—Cl)
605	ν(Mn—O)
793	δ(COO)
1312, 1409	ν_s_(COO)
1657	ν_as_(COO)
2352	ν(N—H⋯O)
3182	ν_s_(NH_2_)
3390	ν(OH)(H_2_O) / ν_as_(NH_2_)

**Table 3:** UV–Vis data (nm) for (I)

Wavelength	Assignment
206	π→π*
240	MLCT
329	*n*→π*

**Table 4:** Experimental details

Crystal data	
Chemical formula	(CH_6_N_3_)[Mn(C_2_O_4_)Cl(H_2_O)]·H_2_O
*M*_r_	274.53
Crystal system, space group	Triclinic, *P* 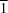
Temperature (K)	298
*a*, *b*, *c* (Å)	6.740 (5), 7.514 (7), 9.810 (2)
α, β, γ (°)	84.46 (3), 78.15 (4), 88.57 (6)
*V* (Å^3^)	484.0 (6)
*Z*	2
Radiation type	Mo *K*α
μ (mm^−1^)	1.65
Crystal size (mm)	0.50 × 0.43 × 0.34

Data collection	
Diffractometer	Enraf–Nonius CAD-4
Absorption correction	ψ scan (North *et al.*, 1968 ▸)
*T*_min_, *T*_max_	0.551, 0.718
No. of measured, independent and observed [*I* > 2σ(*I*)] reflections	4226, 2114, 2018
*R*_int_	0.016
(sin θ/λ)_max_ (Å^−1^)	0.638

Refinement	
*R*[*F* ^2^ > 2σ(*F* ^2^)], *wR*(*F* ^2^), *S*	0.024, 0.065, 1.10
No. of reflections	2114
No. of parameters	155
No. of restraints	11
H-atom treatment	H-atom parameters not refined
Δρ_max_, Δρ_min_ (e Å^−3^)	0.39, −0.30

Computer programs: *CAD-4 EXPRESS* (Duisenberg, 1992 ▸), *XCAD4* (Harms & Wocadlo, 1995 ▸), *SHELXS97* (Sheldrick, 2008 ▸), *SHELXL2014* (Sheldrick, 2015 ▸), *DIAMOND* (Brandenburg, 2006 ▸), *WinGX* (Farrugia, 2012 ▸) and *publCIF* (Westrip, 2010 ▸).

## supplementary crystallographic information

### Crystal data

**Table:**

(CH_6_N_3_)[Mn(C_2_O_4_)Cl(H_2_O)]·H_2_O	*Z* = 2
*M**_r_* = 274.53	*F*(000) = 278
Triclinic, *P*1	*D*_x_ = 1.884 Mg m^−^^3^
*a* = 6.740 (5) Å	Mo *K*α radiation, λ = 0.71073 Å
*b* = 7.514 (7) Å	Cell parameters from 25 reflections
*c* = 9.810 (2) Å	θ = 10–15°
α = 84.46 (3)°	µ = 1.65 mm^−^^1^
β = 78.15 (4)°	*T* = 298 K
γ = 88.57 (6)°	Prism, colourless
*V* = 484.0 (6) Å^3^	0.50 × 0.43 × 0.34 mm

### Data collection

**Table:**

Enraf–Nonius CAD-4 diffractometer	*R*_int_ = 0.016
Radiation source: fine-focus sealed tube	θ_max_ = 27.0°, θ_min_ = 2.1°
ω/2θ scans	*h* = −8→8
Absorption correction: ψ scan (North *et al.*, 1968)	*k* = −9→9
*T*_min_ = 0.551, *T*_max_ = 0.718	*l* = −12→12
4226 measured reflections	2 standard reflections every 120 reflections
2114 independent reflections	intensity decay: 1.4%
2018 reflections with *I* > 2σ(*I*)	

### Refinement

**Table:**

Refinement on *F*^2^	Primary atom site location: structure-invariant direct methods
Least-squares matrix: full	Secondary atom site location: difference Fourier map
*R*[*F*^2^ > 2σ(*F*^2^)] = 0.024	Hydrogen site location: inferred from neighbouring sites
*wR*(*F*^2^) = 0.065	H-atom parameters not refined
*S* = 1.10	*w* = 1/[σ^2^(*F*_o_^2^) + (0.034*P*)^2^ + 0.1929*P*] where *P* = (*F*_o_^2^ + 2*F*_c_^2^)/3
2114 reflections	(Δ/σ)_max_ = 0.001
155 parameters	Δρ_max_ = 0.39 e Å^−^^3^
11 restraints	Δρ_min_ = −0.30 e Å^−^^3^

### Special details

**Table:**

Geometry. All esds (except the esd in the dihedral angle between two l.s. planes) are estimated using the full covariance matrix. The cell esds are taken into account individually in the estimation of esds in distances, angles and torsion angles; correlations between esds in cell parameters are only used when they are defined by crystal symmetry. An approximate (isotropic) treatment of cell esds is used for estimating esds involving l.s. planes.
Refinement. Refinement of F^2^ against ALL reflections. The weighted R-factor wR and goodness of fit S are based on F^2^, conventional R-factors R are based on F, with F set to zero for negative F^2^. The threshold expression of F^2^ > 2sigma(F^2^) is used only for calculating R-factors(gt) etc. and is not relevant to the choice of reflections for refinement. R-factors based on F^2^ are statistically about twice as large as those based on F, and R- factors based on ALL data will be even larger.

### Fractional atomic coordinates and isotropic or equivalent isotropic displacement parameters (Å^2^)

**Table:**

	*x*	*y*	*z*	*U*_iso_*/*U*_eq_	Occ. (<1)
Mn1	0.72543 (3)	0.49656 (3)	0.22092 (2)	0.02231 (9)	
Cl1	1.03175 (7)	0.65970 (6)	0.23354 (5)	0.03534 (12)	
N2	0.1713 (2)	0.28093 (18)	0.43164 (16)	0.0312 (3)	
H2B	0.1064	0.3297	0.5036	0.037*	
H2A	0.1950	0.3411	0.3509	0.037*	
O4	0.47159 (18)	0.36133 (16)	0.15474 (12)	0.0285 (2)	
O2	0.50953 (17)	0.66001 (14)	0.35754 (11)	0.0247 (2)	
N1	0.3354 (3)	0.03734 (19)	0.33501 (16)	0.0351 (3)	
H1A	0.3768	−0.0715	0.3441	0.042*	
H1B	0.3592	0.0972	0.2542	0.042*	
O3	0.69372 (19)	0.63896 (16)	0.02047 (12)	0.0286 (2)	
O1	0.66931 (18)	0.34160 (15)	0.42455 (11)	0.0268 (2)	
OW1	0.9107 (2)	0.26878 (18)	0.14629 (14)	0.0367 (3)	
HW1	0.854 (3)	0.189 (3)	0.112 (2)	0.055*	
HW2	1.027 (2)	0.290 (3)	0.100 (2)	0.055*	
C1	0.4541 (2)	0.59228 (18)	0.48007 (15)	0.0198 (3)	
N3	0.1977 (2)	0.02218 (19)	0.56968 (16)	0.0326 (3)	
H3A	0.2386	−0.0867	0.5796	0.039*	
H3B	0.1323	0.0721	0.6409	0.039*	
C2	0.4359 (2)	0.41972 (19)	0.03918 (15)	0.0221 (3)	
C3	0.2351 (2)	0.1125 (2)	0.44522 (17)	0.0253 (3)	
OW2	0.7198 (6)	1.0392 (5)	0.0066 (7)	0.0831 (13)	0.816 (13)
HW3	0.719 (8)	0.9263 (18)	0.025 (5)	0.125*	0.816 (13)
HW4	0.793 (7)	1.067 (6)	−0.074 (3)	0.125*	0.816 (13)
OW2B	0.712 (2)	0.9962 (16)	0.089 (2)	0.061 (5)	0.184 (13)
HW5	0.637 (17)	0.955 (12)	0.039 (9)	0.091*	0.184 (13)
HW6	0.79 (2)	0.912 (12)	0.115 (14)	0.091*	0.184 (13)

### Atomic displacement parameters (Å^2^)

**Table:**

	*U*^11^	*U*^22^	*U*^33^	*U*^12^	*U*^13^	*U*^23^
Mn1	0.02388 (14)	0.02454 (14)	0.01771 (13)	−0.00049 (9)	−0.00325 (9)	0.00002 (9)
Cl1	0.0292 (2)	0.0383 (2)	0.0387 (2)	−0.00641 (17)	−0.00517 (17)	−0.00671 (17)
N2	0.0365 (8)	0.0223 (6)	0.0341 (7)	0.0016 (6)	−0.0084 (6)	0.0032 (5)
O4	0.0324 (6)	0.0304 (6)	0.0219 (5)	−0.0077 (5)	−0.0073 (5)	0.0071 (4)
O2	0.0291 (6)	0.0231 (5)	0.0197 (5)	0.0029 (4)	−0.0024 (4)	0.0026 (4)
N1	0.0438 (9)	0.0241 (7)	0.0340 (8)	0.0004 (6)	−0.0024 (7)	0.0019 (6)
O3	0.0299 (6)	0.0319 (6)	0.0242 (5)	−0.0109 (5)	−0.0080 (5)	0.0039 (4)
O1	0.0327 (6)	0.0224 (5)	0.0217 (5)	0.0075 (4)	0.0008 (4)	0.0007 (4)
OW1	0.0333 (7)	0.0356 (7)	0.0377 (7)	0.0004 (5)	0.0032 (6)	−0.0080 (5)
C1	0.0211 (7)	0.0174 (7)	0.0208 (7)	−0.0008 (5)	−0.0048 (5)	0.0000 (5)
N3	0.0401 (8)	0.0230 (7)	0.0332 (7)	0.0021 (6)	−0.0074 (6)	0.0036 (6)
C2	0.0231 (7)	0.0222 (7)	0.0194 (7)	−0.0006 (6)	−0.0015 (6)	0.0000 (5)
C3	0.0244 (7)	0.0202 (7)	0.0326 (8)	−0.0043 (6)	−0.0101 (6)	0.0019 (6)
OW2	0.123 (3)	0.0459 (16)	0.076 (3)	−0.0154 (15)	−0.002 (2)	−0.0180 (19)
OW2B	0.078 (8)	0.031 (5)	0.067 (11)	−0.012 (5)	0.009 (6)	−0.020 (6)

### Geometric parameters (Å, º)

**Table:**

Mn1—O1	2.1798 (14)	O1—C1^ii^	1.251 (2)
Mn1—OW1	2.187 (2)	OW1—HW1	0.846 (9)
Mn1—O3	2.1936 (13)	OW1—HW2	0.835 (9)
Mn1—O2	2.2024 (18)	C1—O1^ii^	1.251 (2)
Mn1—O4	2.2476 (19)	C1—C1^ii^	1.552 (3)
Mn1—Cl1	2.4581 (19)	N3—C3	1.318 (2)
N2—C3	1.329 (2)	N3—H3A	0.8600
N2—H2B	0.8600	N3—H3B	0.8600
N2—H2A	0.8600	C2—O3^i^	1.257 (2)
O4—C2	1.2434 (19)	C2—C2^i^	1.547 (3)
O2—C1	1.2451 (19)	OW2—HW3	0.850 (10)
N1—C3	1.321 (2)	OW2—HW4	0.849 (10)
N1—H1A	0.8600	OW2B—HW5	0.851 (10)
N1—H1B	0.8600	OW2B—HW6	0.856 (10)
O3—C2^i^	1.257 (2)		
			
O1—Mn1—OW1	85.68 (7)	C3—N1—H1B	120.0
O1—Mn1—O3	164.34 (5)	H1A—N1—H1B	120.0
OW1—Mn1—O3	100.06 (7)	C2^i^—O3—Mn1	116.88 (11)
O1—Mn1—O2	75.35 (7)	C1^ii^—O1—Mn1	116.15 (10)
OW1—Mn1—O2	160.68 (5)	Mn1—OW1—HW1	117.6 (17)
O3—Mn1—O2	97.39 (6)	Mn1—OW1—HW2	117.3 (17)
O1—Mn1—O4	92.12 (7)	HW1—OW1—HW2	111.4 (15)
OW1—Mn1—O4	85.45 (8)	O2—C1—O1^ii^	126.35 (14)
O3—Mn1—O4	73.99 (6)	O2—C1—C1^ii^	117.50 (16)
O2—Mn1—O4	91.49 (7)	O1^ii^—C1—C1^ii^	116.15 (16)
O1—Mn1—Cl1	100.46 (6)	C3—N3—H3A	120.0
OW1—Mn1—Cl1	90.50 (7)	C3—N3—H3B	120.0
O3—Mn1—Cl1	94.08 (6)	H3A—N3—H3B	120.0
O2—Mn1—Cl1	96.47 (7)	O4—C2—O3^i^	126.48 (15)
O4—Mn1—Cl1	166.46 (3)	O4—C2—C2^i^	116.80 (17)
C3—N2—H2B	120.0	O3^i^—C2—C2^i^	116.73 (16)
C3—N2—H2A	120.0	N3—C3—N1	120.57 (16)
H2B—N2—H2A	120.0	N3—C3—N2	119.27 (16)
C2—O4—Mn1	115.50 (11)	N1—C3—N2	120.16 (16)
C1—O2—Mn1	114.82 (10)	HW3—OW2—HW4	109.7 (17)
C3—N1—H1A	120.0	HW5—OW2B—HW6	108.7 (18)

Symmetry codes: (i) −*x*+1, −*y*+1, −*z*; (ii) −*x*+1, −*y*+1, −*z*+1.

### Hydrogen-bond geometry (Å, º)

**Table:**

*D*—H···*A*	*D*—H	H···*A*	*D*···*A*	*D*—H···*A*
N1—H1*A*···O2^iii^	0.86	2.19	3.047 (3)	175
N1—H1*B*···O4	0.86	2.20	2.917 (3)	140
N2—H2*A*···Cl1^iv^	0.86	2.85	3.509 (3)	135
N2—H2*B*···Cl1^ii^	0.86	2.56	3.357 (2)	154
N2—H2*A*···O4	0.86	2.38	3.054 (3)	135
N3—H3*A*···O1^v^	0.86	2.00	2.854 (3)	172
N3—H3*B*···Cl1^ii^	0.86	2.57	3.363 (3)	154
O*W*1—H*W*1···O*W*2^iii^	0.85 (1)	1.96 (1)	2.793 (4)	170 (3)
O*W*1—H*W*1···O*W*2*B*^iii^	0.85 (1)	1.82 (2)	2.643 (11)	165 (3)
O*W*1—H*W*2···O3^vi^	0.84 (1)	2.06 (1)	2.890 (3)	176 (3)
O*W*2—H*W*3···O3	0.85 (1)	2.17 (2)	3.005 (5)	165 (5)
O*W*2—H*W*4···Cl1^vii^	0.85 (1)	2.61 (3)	3.319 (6)	142 (4)
O*W*2*B*—H*W*5···O3	0.85 (1)	2.42 (11)	2.840 (10)	112 (9)
O*W*2*B*—H*W*6···Cl1	0.86 (1)	2.81 (1)	3.656 (18)	172 (11)

Symmetry codes: (ii) −*x*+1, −*y*+1, −*z*+1; (iii) *x*, *y*−1, *z*; (iv) *x*−1, *y*, *z*; (v) −*x*+1, −*y*, −*z*+1; (vi) −*x*+2, −*y*+1, −*z*; (vii) −*x*+2, −*y*+2, −*z*.
